# The importance of rotation to teach secure half-hitch sequences in surgery

**DOI:** 10.52054/FVVO.15.4.101

**Published:** 2023-12-13

**Authors:** A Romeo, I Cipullo, W Kondo, C Benedetto, B Amro, A Ussia, A Wattiez, P.R. Koninckx

**Affiliations:** Project Leader Research Educational Center University of Turin; Department Obstetrics and Gynecology Ospedale Regina Montis Regalis di Mondovìn, Turin, Italy; Centro Avançado de Cirurgia Ginecológica, Curitiba, Brazil; Latifa Hospital, Dubai, United Arab Emirates; Gruppo Italo Belga, Villa Del Rosario, Rome, Italy; University of Strasbourg, Strassbourg, France; Prof emeritus Obstetrics and Gynecology KULeuven, Leuven, Belgium, University of Oxford, Oxford, UK, University Cattolica, Rome, Italy and Moscow State University, Moscow, Russia

**Keywords:** Knot sequences, half-knots, half-hitches, knot rotation, knot security, loop security

## Abstract

**Background:**

Knot security of half-knot (H) sequences varies with rotation, but half-knots risk destabilisation.

**Objectives:**

To investigate the rotation of half-hitch (S) sequences on knot security.

**Materials and Methods:**

The loop and knot security of symmetrical and asymmetrical sliding and blocking half-hitch sequences was measured using a tensiometer.

**Results:**

Loop security of symmetrical sliding half-hitch sequences is much higher than asymmetrical sequences, increasing from 6+2 to 21+2 and from 27+6 to 48+5 Newton (N) for 2 and 4 half-hitches respectively (both P<0.0001). Symmetrical sliding sequences are more compact and remain in the same plane, squeezing the passive thread, while asymmetrical sequences rotate loosely around the passive end. Blocking sequences are superior when asymmetrical since changing the passive end acts like changing rotation, transforming the asymmetrical sliding into a symmetrical blocking half-hitch on the new passive thread. The knot security of 2 sliding and 1 blocking half-hitch doubles from 52+3 to 98+2 N for the worst (asymmetric sliding and symmetric blocking, SSaSsb) or best rotation sequences (SSsSab). Adding a second asymmetric blocking half-hitch (Sab) increases security further to 105+3 N. The overall knot security of four-throw, correctly rotated, half-hitch (SSsSabSab) or half-knot (H2H1sH1s, H2H2a and H2H2s) sequences is similar for four suture diameters.

**Conclusion:**

Rotation affects the security of half-hitch sequences, which should be symmetrical when sliding, and asymmetrical when blocking.

**What is new?:**

Half-hitch sequences are clinically superior to half-knot sequences. They do not risk destabilisation, and loop security improves approximation of tissues under traction, permitting tight knots.

## Introduction

Suturing and knot tying are basic skills in surgery, but knot stability has been poorly investigated. The methods and material for suturing used to be a matter of habit, guesswork or tradition (Haxton, 1965) until the tensiometer (Thacker et al., 1975; Tera and Aberg, 1976; Trimbos, 1984; Trimbos et al., 1986; Trimbos et al., 1991) was introduced to measure knot security, defined as the resistance to sliding to open with increasing forces. Later loop security was defined as the resistance to sliding of the first loop for half-knots or sliding half-hitches. Loop security indicates the forces that maintain approximation until subsequent blocking knots are added (Trimbos, 1984; Burkhart et al., 2000). Loop security is important for making tight knots, notwithstanding much traction on the edges. The forces needed after suturing a bowel or vaginal cuff are not documented in abdominal and gynaecological surgery. However, common sense indicates that during coughing, forces on the fascia following abdominal surgery or on the promontory after promontofixation are much higher than those needed for bowel or vaginal cuff suturing. Also, knots that slide to open with forces less than 5 or 10 N might be dangerous (Romeo et al., 2020).

In surgery, knot security varies with the type of suture and the knot characteristics but not with suture size (Silver et al., 2016). In surgery, most knots (for an overview (Chisnall, 2020)) are sequences of half-hitches (S) or half-knots (H) (Table I). Half-hitches are made by pulling one end of the suture, called the passive end. Half-knots (H) require that both ends are pulled symmetrically in the opposite direction as entering the knotting loop. Half-hitches consist of 1 throw and half-knots of 1 or more throws. Clockwise or counterclockwise rotation for the first half-knot or half-hitch is not important. For subsequent half- knots or half-hitches, rotation in comparison with the previous one is important. For half-knots, an opposite rotation of the active end around the passive results in a flat square knot, which is visually recognised as symmetrical (Romeo et al., 2020). Rotation used to be indicated by = for symmetrical, x for asymmetrical and blocking half-hitches by //. However, symmetrical rotation was called identical, and the indication of the type of knot was variable. For example, a 1-throw flat square knot could be indicated as 1=1 or H=H (Table I) (Burkhart et al., 2000). To avoid ambiguity, we prefer to indicate the type (‘H’ for half-knots and ‘S’ for half-hitches), followed by the number of throws and the rotation (‘s’ for symmetric and ‘a’ for asymmetric), followed for half-hitches by a ‘b’ if blocking. Sliding cinch knots (Mackenzie, 2012) that can be blocked when in place, such as the Röder knot, will not be discussed.

**Table I:** Knot sequences used to be indicated with = for symmetrical (called identical), x for asymmetrical and // for blocking. We prefer a more intuitive indication, with the type of each half-knot (H) or hitch (S), followed by the rotation around the passive thread in comparison with the previous one, using ‘s’ for symmetrical (alternate rotation around the passive end by mono manual suturing) and ‘a’ for asymmetrical (similar rotation around the passive tread). Finally, whether half-hitches are sliding (s) or blocking (b) is indicated. For half-hitches, the number of throws is not indicated since always one, and sliding is not indicated to avoid confusion with the s of symmetric. A 2-throw half-hitch, resulting from the transformation of a 2-throw half-knot, always ends with an insecure knot.

Knots	Throws	Knot Sequences		Older Indication	New Indication
Half knot	1,2,3				H1, H2, H3
		2nd symmetric		H=H, 1=1	H1H1s, H2H1s, H2H2s, H3H2s
		2nd asymmetric		HxH, 1x1	H1H1a, H2H1a, H2H2a, H3H2a
Secure half-knot sequences: H2H1sH1s, H2H2s or H2H2a, H3H2s or H3H2a					
Half hitch	1,2				S(1), (S2)
		2nd symmetric		S=S, 1=1	SSs,
			and sliding or blocking	S=S or S//S	SSs(s), SSsb
		2nd asymmetric		SxS, 1x1	SSa
			and sliding or blocking	SxS or S//xS	SSa(s), Ssab
Secure Half-hitch sequences: SSsSabSab					

Clockwise or counterclockwise rotation is obvious to the surgeon. With mono-manual knot tying, an alternate rotation of the active end around the passive results in a symmetrical flat square knot or two symmetrical half-hitches. Similar rotation results in an asymmetric flat square knot or two asymmetric half-hitches. With bimanual suturing, changing the active and passive ends, similar rotation results in a symmetrical knot. Rotation is important for the strength of half-knot and half- hitch sequences, symmetrical sequences being much more resistant to opening than asymmetrical ones (Trimbos, 1984; Schubert et al., 2002; Van Leeuwen and Trimbos, 2012; Romeo et al., 2018; Romeo et al., 2020) for several sizes of surgical sutures (Ivy et al., 2004). This is not surprising considering the geometry of the knots: two symmetrical half-knots, such as H1H1s, have the tails entering and going out of the knot in the same plane. Also, symmetrical half-hitches remain in the same plane and are more compact than asymmetrical ones. For half-knots, the effect of the rotation sequence varies with the number of throws. The one throw H1H1s is much more stable than H1H1a, the H2H1s is better than H2H1a, H2H2s and H2H2a are pretty similar, while the H3H2a is slightly more stable than H3H2s. (Kondo et al., 2018; Romeo et al., 2018; Romeo et al., 2020). The effect of rotation for blocking sequences has not been described.

If one end is pulled, a half-knot can be transformed into a half-hitch, an H1 becoming an S1 and an H2 an S2. Unfortunately, this can occur unpredictably during knot tying, when the first half- knot is destabilised because of insufficient loop security or by a small mistake during suturing, such as inadvertently pulling one end. The risk of destabilisation is high during laparoscopic suturing when sutures are short or the surgeon is less experienced. This transformation of half-knots into half-hitches results in decreased knot security with an S2 resulting invariably in a poor knot. This unintended or accidental reorganisation explains that approximately 5% of surgical knots, such as H2H1sH1s with a double throw half-knot followed by two symmetrical single throw half-knots, occasionally opened with little tension of less than 5N (Romeo et al., 2018; Romeo et al., 2020).

Gynecologic surgery often requires suturing deep in the pelvis, making perfect half-knots difficult because of poorly recognised low loop security and since the need for inverting the pulling direction for subsequent knots risks destabilising the first knot. Sequences of half-hitches might therefore be more suited for deep suturing. Intrigued by the reorganisation of half-knots and the importance of rotation for half-knot sequences, we decided to investigate the effect of rotation on the knot and loop security for sliding and blocking half-hitch sequences.

## Materials and Methods

### Aim of the Study, Knot Combinations and Power Estimations

This study aimed to investigate the effect of rotation on half-hitch knot combinations and, more specifically blocking sequences. A first experiment evaluated differences in forces needed to slide open of 2 and 4, symmetrical and asymmetrical, sliding half-hitch sequences in a factorial design using Poliglactin (Polisorb) 0. A second experiment evaluated the addition of a blocking half-hitch to 2 sliding half-hitches in a factorial design using symmetrical and asymmetrical sequences for the second sliding half-hitches and the third blocking half-hitches. Subsequently, the effect of adding a second blocking half-hitch was evaluated. Finally, the knot security of the best four-throw half- hitch combination (SSsSabSab) was compared to half-knot combinations with a high knot security breaking or close to breaking the suture, such as the four-throw surgical knots, H2H1SH1s, H2H2a and H2H2s using four diameters of vicryl, i.e. 3-0, 2-0, 0 and 1.

In each experiment, all knots were made by the same experienced person (AR) and knot tying was block randomised by group. Thus for four groups, 1 knot of each group was made before starting the second series. All knots were subsequently tested together since the results of 10 H2H1sH1s knots, with polyglactin 0, were similar when tested immediately: (97.3+4.3 N), after one day (95.1+5.3 N) and after 14 days (102.6+3.5 N).

Institutional review board (IRB) approval was not needed for experiments in vitro not involving humans or animals, as confirmed in writing by the IRB of Leuven University.

### Knot Classification, knot tying and testing

Knot classification, tying, and testing were described in detail previously (Kondo et al., 2018; Romeo et al., 2018; Romeo et al., 2020). A knot is defined by an ‘H’ for half-knots or ‘S’ for half-hitches), followed by the number of throws, and the rotation (‘s’ for symmetric and ‘a’ for asymmetric in comparison with the previous one ), followed for half-hitches by ‘s’ for sliding and ‘b’ for blocking. However, for half-hitches, the number of throws, since normally 1, and sliding, can be omitted to avoid confusion with the symmetric. With mono-manual suturing, an opposite rotation of the active end results in a symmetrical (s) sequence and a similar rotation in an asymmetrical (a) sequence. Half-hitches are sliding as long as the passive end of the suture remains the same and become blocking when the active and passive ends are switched. To avoid confusion between symmetrical (s) and sliding, only blocking (b) is indicated. Thus, SSsSsbSab indicates a second symmetrical and sliding, a third symmetrical and blocking and a fourth asymmetrical and blocking half-hitch.

Standardised laparoscopic knots were made as described, using Romeo’s gladiator knot-tying technique (Liceaga et al., 2013) in a laparoscopic simulator using Karl Storz Full HD Imaging System (IMAGE 1 HUB HD and a 3-chip HD camera head) and 2 Karl Storz needle holders (KARL STORZ KOH Macro Needle Holder)(Kondo et al., 2018). Sutures of 18 cm were tied around a 15-mm plastic tube using the different knot combinations to be evaluated. After knot tying, the suture threads were cut at exactly 10 mm to permit the detection of some sliding. These loops were subsequently mounted on the hooks of a digital dynamometer Sauter FH 500 capacity 500 NW and tested at a 200 mm/min speed. With increasing forces, the knot combination slipped to open or blocked, causing the suture to break. The two endpoints thus are breaking or sliding to open and the force (N) at which the knot slips to open or breaks. Testing was done at the Research Educational Centre of Turin University.

## Statistics

Statistical evaluation was done with SAS (Inc, 2020) and means and SDs are given unless indicated otherwise. A factorial design with 10 knots for each group thus resulted in a statistical power of 40 knots (Armitage and Berry, 1987).

Differences were evaluated with Wilcoxon signed rank test. Analysis of the factorial design was performed using a 2-way analysis of variance for non-Gaussian distributions (proc GLM).

## Results

**Figure 1:**
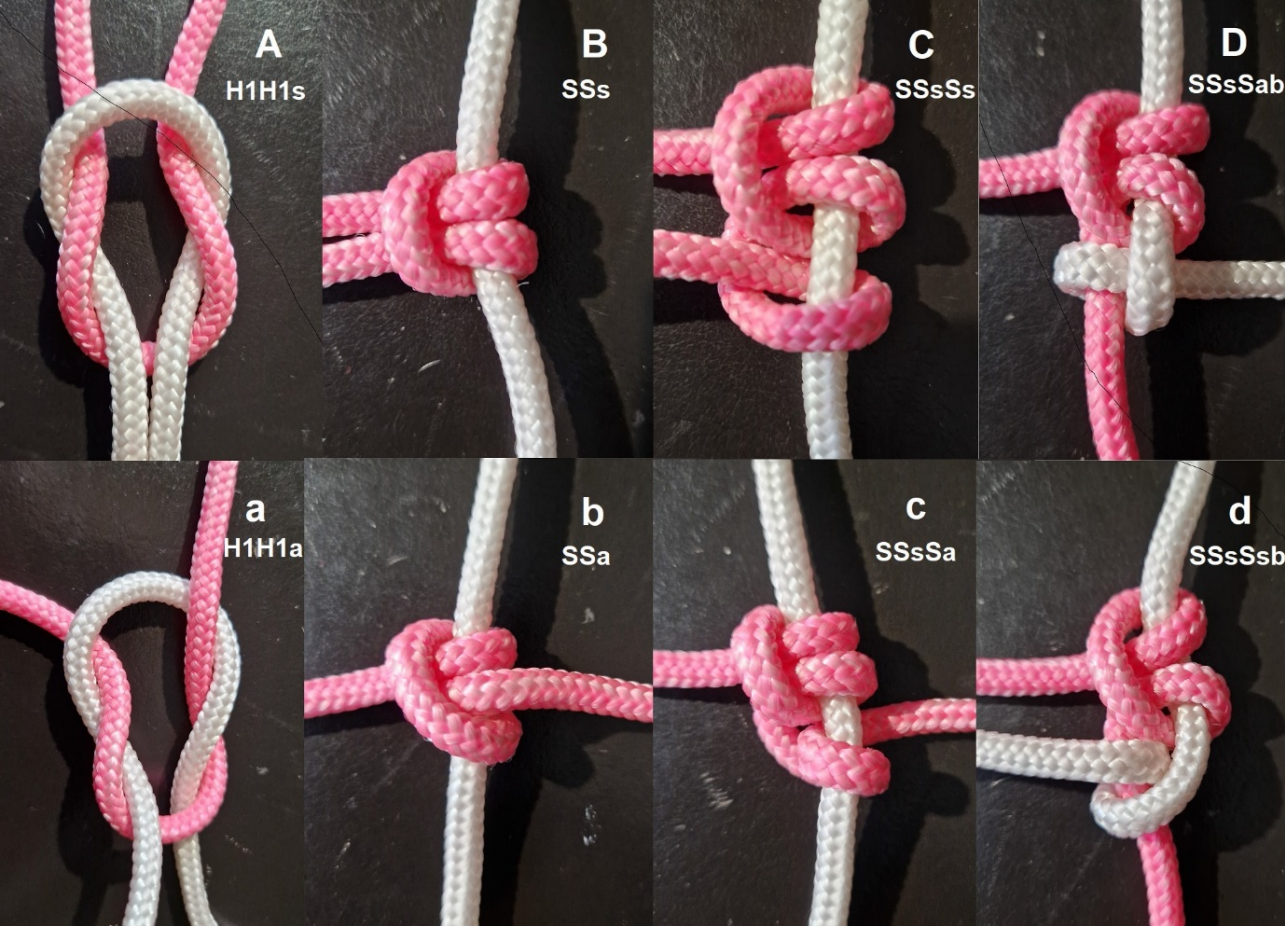
Macroscopic evaluation of 2 symmetric half-knots (A), which transform into symmetrical half-hitches (B), the active red end squeezing the passive white. When two asymmetric half-knots (a) are transformed into asymmetric half- hitches (b), the active red end runs around the passive white ending in the opposite direction. Picture C shows that three symmetric sliding half-hitches (SSsSs) are composed of twice SSs, each squeezing the passive white thread with the active ends on one side. When the red active end of the last half-hitch is pulled, the red becomes the passive end, transforming the symmetrical sliding half-hitch into an asymmetrical blocking half-hitch with the new passive white end pointing in the opposite direction. Pictures c and d illustrate how the third asymmetric sliding half-hitch (SssSa) transforms into a symmetric blocking half-hitch (SSsSsb), the red and white ends now squeezing the passive red like two symmetric half- hitches.

Macroscopically, a sequence of symmetrical sliding half-hitches (alternating rotation) is more compact than an asymmetrical series (4cm for length for five symmetrical half-hitches versus 5.5 cm for five asymmetrical half-hitches, using the 4mm thread of Figure 1). Similarly, asymmetrical blocking half-hitches are more compact than symmetrical ones (Figure 1). Symmetrical sliding (Ss) and asymmetrical blocking (Sab) half-hitches remain in the same plane, whereas series of Sa or Ssb rotate more loosely around the passive thread.

**Figure 2:**
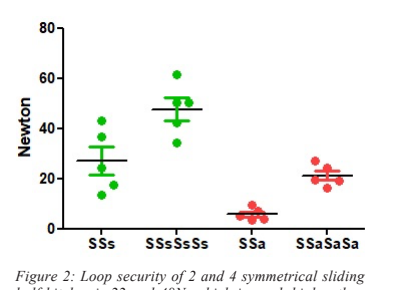
Loop security of 2 and 4 symmetrical sliding half-hitches is 22 and 48N, which is much higher than 2 or 4 asymmetrical ones. (twice P<0.0001). Loop security also increases with the number of half-hitches boh for symmetrical and asymmetrical sequences (twice P<0.0001).

Comparing 2 and 4, symmetrical and asymmetrical sliding half-hitches, the loop security or the resistance to sliding open, increases with the number of half-hitches (P<0.0001) and when half-hitches are symmetrical (P<0.0001)( two-way analysis of variance). For Polyglactin 0, opening forces of 2 and 4 asymmetrical and symmetrical half-hitches increase from 6+2 and 21+2 to 27+6 and 48+5 N (Figure 2).

**Figure 3:**
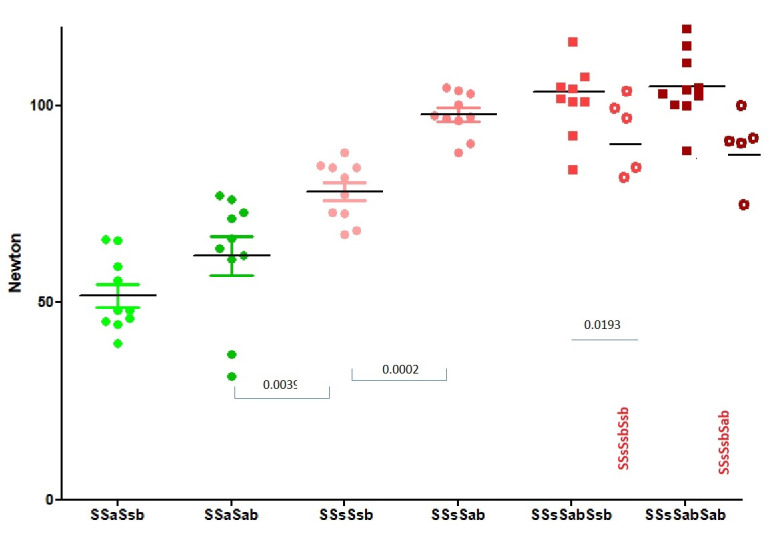
The knot security of 2 sliding and one blocking half-hitch demonstrates the superiority of symmetrical sliding (P<0.0001) half-hitches and asymmetrical blocking half-hitches(P<0.0001). Adding a second blocking half-hitch improves knot security further, confirming the superiority of asymmetric blocking half-hitches. With a second asymmetric blocking half-hitch (SSsSabSab), all sutures break; with a second symmetrical blocking half-hitch (SSsSabSsb), 8/10 slide to open (P= 0.0007). A second asymmetric blocking half-hitch cannot compensate for a first symmetric blocking half-hitch.

A sequence of 2 sliding and 1 blocking half- hitch (Figure 3) confirms by two-way analysis of variance that the knot security is better when the second sliding half-hitch is symmetrical (P<0.0001) and the third blocking half-hitch asymmetrical (P<0.0001). The knot security doubles from 52+3N to 98+2 N forn the worst (SSaSsb) to the best rotation sequences (SSsSab). When, to the best combination SSsSab, a second asymmetrical blocking half-hitch is added, knot security increases further to 105+3 N. Although this increase is not statistically significant, a second asymmetric blocking half-hitch (Sab) results in broken sutures for all knots, whereas after adding a symmetric Ssb, 8/10 sutures slide to open (p=0.0007; chi-square). However, a second correct Sab cannot compensate for a first wrong Ssb: all SSsSsbSab knots slide to open compared to none of SSsSabSab knots (P=0.0003).

The knot security of a correct sequence SSsSabSab is comparable to the best half-knot sequences H2H1SH1s, H2H2a and H2H2s for sizes of 3-0, 2-0, 0, and 1 vicryl. Although clinically not meaningful, by 2-way analysis of variance knot security of SSsSabSab is slightly less than H2H2a (P<0.0001), H2H2s (P=0.0001) and H2H1sH1s (P=0.0002. ); H2H2a is better than H2H2s or H2H1sH1s (twice P<0.0001); H2H2s and H2H1sH1s are not different. However, with all knot combinations and all sizes tested, occasionally, knots slide to open instead of breaking the suture, albeit with forces close to the breaking force of the suture.

### Comment

Previous observations that rotation of sequential half-knots and half-hitches are important for knot security (Tera and Aberg, 1976; Trimbos, 1984; Romeo et al., 2018; Romeo et al., 2020) are confirmed. New is that rotation is similarly important for blocking half-hitches, which is logical since changing the active and passive ends has a similar effect on the knot structure as changing rotation.

Our data confirm that symmetrical sliding half-hitches and half-knots, made by alternating rotation of the active end, are more secure than asymmetrical ones (Tera and Aberg, 1976; Trimbos, 1984; Romeo et al., 2018; Romeo et al., 2020). This is explained by the configuration or 3D structure of these knots. In symmetrical half- knots, the entry and exit of both ends are in the same plane, squeezing the other end under traction. In symmetrical sliding half-hitches, the entry and exit of the active threads around the passive end are in the same direction strangulating the passive thread (Figure 1). In asymmetric half-hitches, the active thread turns around the passive thread, and entry and exit are in the opposite direction resulting in a less compact and, therefore, less secure knot.

A sliding half-hitch is transformed into a blocking half-hitch by pulling the active end, which becomes the new passive end. This changes the rotation of the new active suture around the new passive suture, similar to changing the rotation: an SSs sequence thus transforms into an SSab and an SSa into an SSsb sequence on the new passive thread. This lets us understand that similar to symmetrical sliding half-hitches being more secure, asymmetrical sliding half-hitches, before changing passive ends, are more secure since they transform into symmetrical blocking half-hitches around the new passive thread. It is important to understand the similarity between an asymmetric half-hitch (by similar rotation) and then changing the active and passive ends, and first changing the active and passive ends followed by a symmetrical half-hitch with similar rotation. The former is typically mono-manual suturing, and the latter bimanual suturing.

Clinically a correct rotation of half-hitch sequences is important for loop and knot security. Loop security of sliding half-hitches doubles when symmetric in comparison with asymmetric. Also, knot security of 2 sliding and 1 blocking half- hitch doubles from the worst (SSaSsb) to the best combination (SSsSab). Since half-hitch sequences do not risk destabilisation, half-hitch sequences are more reliable than half-knot sequences, with occasional low knot security (Romeo et al., 2018; Romeo et al., 2020). A high knot security and reproducibility permits using thinner sutures and cutting the tails shorter, thus reducing the mass of suture material with less postoperative adhesion formation (Trew et al., 2011). Finally, if a knot has high reliability and security with breaking or near-breaking forces of the suture, the final knot security cannot improve by adding more half- knots or hitches. It only increases the suture mass and, thus, adhesions.

Rotation is important for teaching knot tying. The passive suture remains unchanged for most surgeons using mono manual suturing for half- knots and half-hitches. They should only pay attention to use alternating rotation for half-knots and sliding half-hitches. Only for blocking half- hitches rotation should be similar.

**Figure 4:**
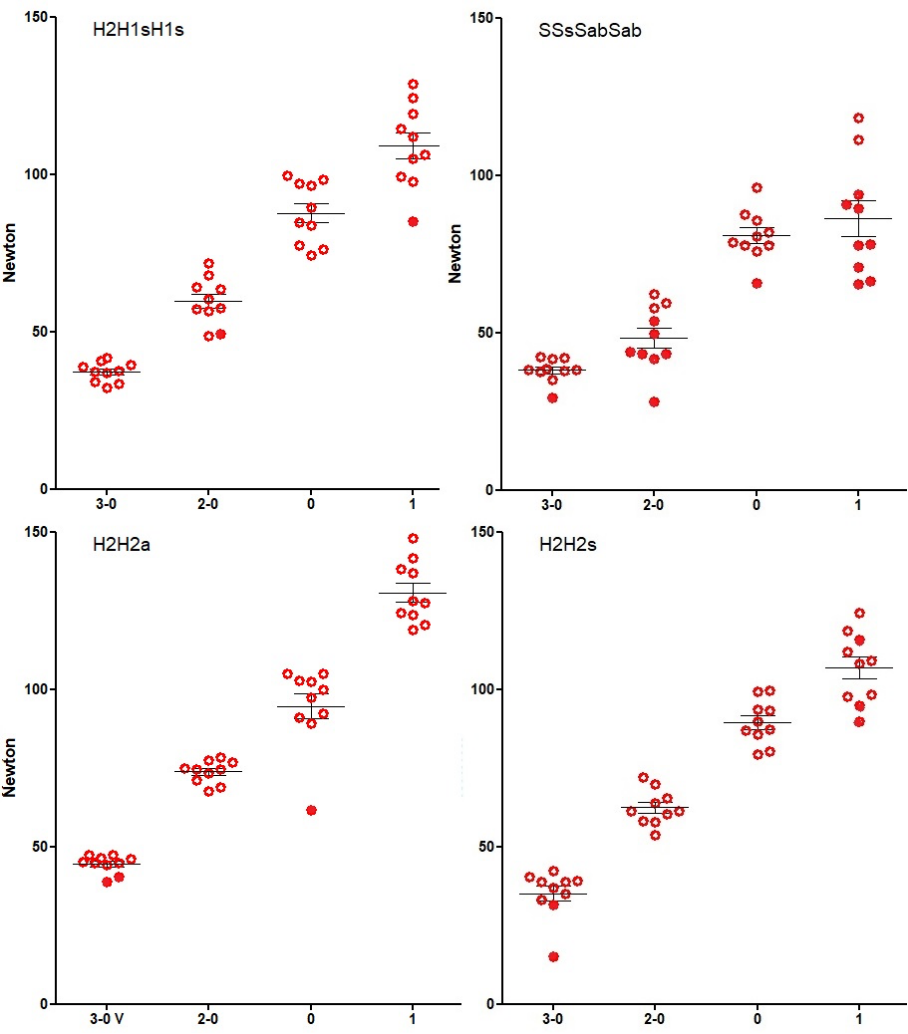
Knot security of correct sequences of 2 symmetrical sliding and two asymmetrical blocking half-hitches(SSsSabSab) compared with correct four-throw half-knots (H2H1sH1s, H2H2a and H2H2s). Open circles indicate the breaking of sutures, and closed circles knots that slide open. Mean, and SEM is shown. By 2-way analysis of variance knot security of SSsSabSab is slightly less than H2H2a (P<0.0001), H2H2s (P=0.0001) and H2H1sH1s (P=0.0002). The knot security of H2H2a is slightly better than H2H2s or H2H1sH1s (twice P<0.0001).

In gynecologic surgery, half-hitch sequences might be preferable to half-knot sequences. First, the 22 N loop security of 2 symmetric half-hitches is slightly superior to the 18 N of a three-throw half-knot. However, if insufficient to keep suture edges approximated, a third sliding half-hitch can be added, increasing loop security to some 45N. The importance of loop security is highlighted by the frequent use of barbed sutures to achieve the same goal during myomectomy (Arena et al., 2021). A second advantage of half-hitch sequences is that the risk of unintended destabilisation is less. Half-knots risk destabilisation by small mistakes transforming a half-knot into a half- hitch. Especially the transformation of an H2 into an S2, results in an unpredictable knot sequence with poor knot security (Romeo et al., 2020). The differences between the knot security of 4 correct half-hitch sequences and correct 4-throw half-knot sequences are clinically not important (Figure 4). We should realise that these knots were made by an experienced and dedicated person (AR) paying attention to the details of rotation and destabilisation. However, in daily clinical surgery, the excellent surgical knot (H2H1sH1s) risks occasionally having low knot security (Romeo et al., 2018) by unnoticed mistakes. Correct half-knot or half-hitch sequences and rotation is expected to be even more important for monofilament sutures, with less friction resistance in the knots. A discussion of barbed sutures and the many cinch knots designed for secure and tight suturing is beyond this manuscript.

## Conclusions

The conclusions for surgery and for teaching knot tying are straightforward. With mono manual suturing, always use alternate rotation (symmetrical) for sliding and similar rotation (asymmetrical) for blocking half-hitches. The loop security of two and certainly three symmetrical sliding half-hitches is superior to the 18N of a 3-throw half-knot and thus preferable to half-knots when forces to keep the edges approximated are strong. The first two or three symmetrical half-hitches, should be secured by two asymmetrical blocking half-hitches resulting in a knot security close to the breaking forces of the suture. Although the knot security of the four throws SSsSabSab is comparable to 4-throw half-knots such as H2H1sH1s or H2H2a or H2H2s, half- hitch sequences do not risk destabilisation during knot tying. Therefore half-hitch sequences are preferable to half-knot sequences, especially when suturing deep in the pelvis with a high risk of destabilisation of half-knots. That some surgeons today use sequences of 6 half-hitches probably reflects clinical prudence when the importance of correct rotation is not fully understood.
